# Integrated genetic and epigenetic prediction of coronary heart disease in the Framingham Heart Study

**DOI:** 10.1371/journal.pone.0190549

**Published:** 2018-01-02

**Authors:** Meeshanthini V. Dogan, Isabella M. Grumbach, Jacob J. Michaelson, Robert A. Philibert

**Affiliations:** 1 Department of Biomedical Engineering, University of Iowa, Iowa City, Iowa, United States of America; 2 Department of Psychiatry, University of Iowa, Iowa City, Iowa, United States of America; 3 Cardio Diagnostics LLC, Coralville, Iowa, United States of America; 4 Department of Internal Medicine, University of Iowa, Iowa City, Iowa, United States of America; 5 Iowa City Veterans Affairs Healthcare System, Iowa City, Iowa, United States of America; 6 Behavioral Diagnostics LLC, Coralville, Iowa, United States of America; Universitatsklinikum Hamburg-Eppendorf, GERMANY

## Abstract

An improved method for detecting coronary heart disease (CHD) could have substantial clinical impact. Building on the idea that systemic effects of CHD risk factors are a conglomeration of genetic and environmental factors, we use machine learning techniques and integrate genetic, epigenetic and phenotype data from the Framingham Heart Study to build and test a Random Forest classification model for symptomatic CHD. Our classifier was trained on n = 1,545 individuals and consisted of four DNA methylation sites, two SNPs, age and gender. The methylation sites and SNPs were selected during the training phase. The final trained model was then tested on n = 142 individuals. The test data comprised of individuals removed based on relatedness to those in the training dataset. This integrated classifier was capable of classifying symptomatic CHD status of those in the test set with an accuracy, sensitivity and specificity of 78%, 0.75 and 0.80, respectively. In contrast, a model using only conventional CHD risk factors as predictors had an accuracy and sensitivity of only 65% and 0.42, respectively, but with a specificity of 0.89 in the test set. Regression analyses of the methylation signatures illustrate our ability to map these signatures to known risk factors in CHD pathogenesis. These results demonstrate the capability of an integrated approach to effectively model symptomatic CHD status. These results also suggest that future studies of biomaterial collected from longitudinally informative cohorts that are specifically characterized for cardiac disease at follow-up could lead to the introduction of sensitive, readily employable integrated genetic-epigenetic algorithms for predicting onset of future symptomatic CHD.

## Introduction

Heart Disease is the leading cause of death in United States.[1] Coronary heart disease (CHD) is the most common type of heart disease. Methods to identify those at risk for sudden death or a myocardial infarction (MI) have been developed. However, their lack in sensitivity have contributed to millions of Americans suffering otherwise preventable cardiac events. In fact, sudden cardiac death is the initial presentation in 15% of patients with CHD.[2, 3] Though clinicians are wary of the potential for cardiac disease at any age, increased attention is paid to individuals with the classic risk factors for CHD defined in the Framingham Heart Study (FHS) including family history of CHD, smoking, elevated systolic blood pressure, diabetes, or anything resembling angina-like chest pain.[4, 5] Depending on the level of suspicion for CHD, the initial examination typically includes a complete physical exam and a fasting lipid panel that includes low density lipoprotein (LDL), high density lipoprotein (HDL) and triglyceride levels.[5] The next level of response is normally an electrocardiogram (ECG) followed by more costly and invasive measures including stress testing and cardiac angiography.[6]

Sadly, most clinically routine tests such as the 12 lead ECG and serum lipid screening are remarkably insensitive for CHD. For example, in a study of 479 patients admitted for acute chest pain with creatine kinase-MB isoenzyme (CK-MB) and troponin T (TnT) confirmed MI, 12 lead ECG was positive in only 33% and 28% of these patients at admission and post-admission, respectively.[7] In the FHS, using a cutoff of 260 mg/dl, elevated serum cholesterol levels performed at intake failed to identify 2/3 of all the males who developed CHD over the subsequent four years. Hence, there is a need for additional methods that could more sensitively diagnose the presence of CHD prior to the occurrence of acute cardiac events.

While a variety of approaches, including imaging, mechanical and bio-electrical techniques have been used [8–10], developing blood based methods has its advantages because of the 1) proof-of-principal provided by prior work with triglycerides and cholesterol, 2) clear involvement of blood components such as platelets and white blood cells in CHD pathogenesis and 3) the ease of integrating blood based approaches into current medical diagnostics.

In the era where the barrier to profile and access omics data is relatively low, the challenge shifts to the mining of more sensitive diagnostic biomarkers. To that end, genome-wide association and exome/genome sequencing studies (see O’Donnell and Nabel, 2011 for review [11]) have been conducted to identify risk associated variations. To date, these studies have isolated approximately 10% of the total genetic risk for CHD.[12, 13] Notably, many of the SNPs noted in these studies map to lipid and inflammation pathways, both of which are known to be important from prior studies of CHD.[13] However, meta-analyses indicate that at best, the contribution of pure genetic approaches to CHD prediction will be minimal.[14] As such, standalone genetic approaches have not been incorporated into routine clinical practice.

Critical to the current work is the observation made in prior smoking associated epigenetic studies. We observed that, smoking associated methylation markers such as the *GPR15* marker cg19859270 [15, 16] did not predict smoking status well in all populations due to confounding of methylation changes by local genetic variation (i.e. gene-environment interactions).[15] This suggested that, accounting for the complex interplay between genetic and environmental factors, latter of which can be measured epigenetically, is vital for increased sensitivity and generalizability in all populations. Incorporating this complexity can drive the discovery of CHD biomarkers forward as it is widely known that CHD is a common complex disease with genetic, environment and gene-environment components.

In that hope, the goal of our study was to demonstrate the viability of an integrated genetic-epigenetic approach in predicting CHD status as an alternative to routinely used conventional CHD risk factors (age, sex, blood pressure, total cholesterol, HDL cholesterol, smoking status and HbA1c to represent diabetes status). Hence, this study was designed to 1) integrate genome-wide genetic and epigenetic data from the FHS obtained using DNA from blood to develop an integrated CHD prediction model, 2) develop a CHD prediction model consisting of conventional risk factors, and 3) compare the CHD prediction performance of these models. While the models in this study are being used to classify individuals based on their current CHD status, they will serve as a proof-of-concept and set the stage for the eventual development of genetic-epigenetic markers for predicting CHD prior to its occurrence (i.e. forecasting).

## Materials and methods

### Ethics statement

This study was conducted using fully anonymized Framingham Heart Study data obtained through dbGAP (https://dbgap.ncbi.nlm.nih.gov). The participants provided written informed consent to a Framingham Heart Study clinical staff to participate in this study. The University of Iowa Institutional Review Board approved all analyses described in this study.

### Framingham Heart Study

The Framingham Heart Study (FHS) (dbGAP study accession: phs000007) has been described in detail elsewhere.[17, 18] The clinical, genetic and epigenetic data included in this study is from the Offspring cohort who 1) attended the eighth examination cycle which was conducted between 2005 and 2008, 2) consented to genetics research, and 3) have peripheral blood genome-wide DNA methylation data.

### Genome-wide DNA methylation

Based on consent and after removing duplicates, DNA methylation data was available for 2,567 individuals. Genome-wide DNA methylation of the Offspring cohort was profiled using the Illumina Infinium HumanMethylation450 BeadChip.[19] (San Diego, CA) The quality control steps have been described in detail in a recent publication.[20]

### Genome-wide genotype

Genome-wide SNP data was profiled using the Affymetrix GeneChip HumanMapping 500K (Santa Clara, CA) array. Of the 2,560 individuals remaining after DNA methylation quality control, 2,406 (1,100 males and 1,306 females) had genotype data. Again, quality control steps have been described in detail in a recent publication.[20] A total of 111 samples were removed after these steps were performed. Of the remaining 2,295 individuals, another 696 individuals were removed due to relatedness (identity by descent >0.1875, which is halfway in between second and third-degree relatives), leaving 1,599 subjects (722 males and 877 females) for further analyses.

### Phenotypes

For each individual, the following data were extracted from the FHS dataset: age, gender, systolic blood pressure (SBP), high-density lipoprotein (HDL) cholesterol level, total cholesterol level, hemoglobin A1C (HbA1c) level, self-reported smoking status, the use of statins, CHD status and date of CHD established. Since DNA methylation was profiled using biomaterial collected during the eighth examination cycle of the Offspring cohort, all phenotype data obtained during this cycle were used in analyses. An individual was categorized as having symptomatic CHD if they received a positive diagnosis from the Framingham Endpoint Review Committee at or prior to their eighth examination cycle (i.e. the CHD established date was equal to or less than their eighth examination cycle date when biomaterial was collected for DNA methylation profiling). This categorization criterion reduced the number of individuals from 1,599 to 1,545 (173 and 1,372 with and without symptomatic CHD, respectively).

### DNA methylation analysis

To identify CHD associated genome-wide DNA methylation changes, linear regression analyses were conducted using data from the 1,545 individuals in the training set in R V3.1.2 as delineated in Eq (1):

$${Meth}_{i}\;\sim \;Age+Gender+Batch+CHD$$ (1)

The association between DNA methylation and CHD was determined while controlling for age, gender and DNA methylation laboratory batch effects. Bonferroni correction for multiple comparisons at a genome-wide α = 0.05 was performed for every regression analysis.[21] A total of 472,822 independent tests were conducted and therefore, only those with a nominal p-value <1e-07 (0.05/472822) were considered to be significantly associated at the genome-wide level.

### Training and testing datasets

As mentioned in the introduction, the goal of this study was to develop an integrated genetic-epigenetic classifier for symptomatic CHD. To achieve this, training and testing datasets were prepared to develop the classifier and subsequently test its predictive capability. The 1,545 individuals (173 and 1,372 with and without symptomatic CHD, respectively) that survived all quality control steps and had a CHD status at their eighth examination cycle were used for variable reduction, variable selection and model training. Since only 173 individuals were diagnosed with CHD, an undersampling approach similar to EasyEnsemble of the majority class was performed to address the class imbalance. [22, 23] Using this approach, eight training sub-datasets were generated, where controls in each subset were randomly undersampled without replacement. This resulted in four training sub-datasets with 171 controls and four others with 172 controls, totaling 1,372 controls.

To assess the predictive capability of the trained model, data from the 696 individuals removed based on their relatedness were used. Again, CHD status and the eighth examination cycle dates of these individuals were compared to categorize them as either positive or negative for symptomatic CHD. From doing so, the number of individuals was reduced from 696 to 669 (314 males and 355 females). Since only 71 of these 669 individuals were diagnosed with symptomatic CHD, the final test dataset was balanced with 71 randomly chosen controls, resulting in the final test dataset of n = 142.

### Variable reduction

The total number of genetic (SNP) and epigenetic (DNA methylation) probes remaining after quality control measures were 403,192 and 472,822, respectively. Due to the large number of variables (876,014 total, excluding phenotypes), we implemented steps as discussed below to reduce the search space and minimized redundancy in the predictors.

Linkage disequilibrium based SNP pruning was performed in PLINK[24] with a window size of 50 SNPs, window shift of 5 SNPs and a pairwise SNP-SNP LD threshold of 0.5. This reduced the number of SNPs from 403,192 to 161,474. To further reduce the number of SNPs, the chi-squared p-value was calculated between the remaining 161,474 SNPs and CHD status. Those with a chi-squared p-value <0.1 were retained for model training, resulting in 17,532 SNPs (~4%).

To reduce the number of DNA methylation loci, first, the correlation was calculated between the 472,822 CpG sites and CHD status. CpG sites were retained if the point bi-serial correlation with CHD was at least 0.1. A total of 138,815 CpG sites remained. Subsequently, Pearson correlation between those 138,815 sites was calculated. If the Pearson correlation between two loci was at least 0.8, the loci with a smaller point bi-serial correlation (i.e. less correlated with CHD) was discarded. In the end, 107,799 DNA methylation loci (~23%) remained for model training.

To systematically feed variables into the classification model, the area under the curve (AUC) of the receiver operating characteristic (ROC) curve of the top 1% methylation and SNPs were calculated and used to rank these loci.

### Model training and testing

Using a stratified 10-fold cross-validation approach, Random Forest (RF)[25] classification models were built independently using *scikit-learn* in Python V2.7.12 [26] on all eight training sub-datasets consisting of genetic, epigenetic and phenotype data. Based on their ROC AUC, loci were fed systematically into the model. Feature importance, accuracy and AUC of RF classifiers were used to select important variables for prediction. A grid search using GridSearchCV was employed to perform 10-fold cross-validation hyper-parameter tuning (maximum features: auto, minimum samples for each split: 2–10, information gain criterion: entropy or gini, maximum tree depth: 500–2500, number of trees: 10000–30000) of the models. The performance metrics of the models were determined. All eight final tuned models were saved for testing on the test dataset where majority voting was used to ensemble the votes of these models.

**Fig 1:**
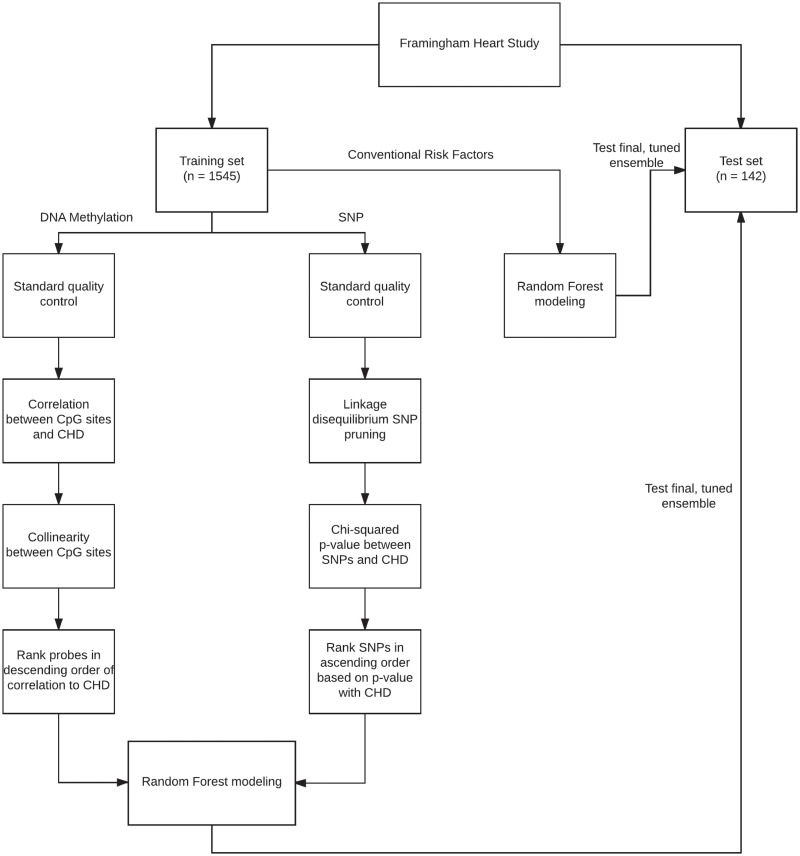
Filtering and modeling. Flowchart summarizing steps performed for Random Forest model training and testing.

To compare the performance of our integrated genetic-epigenetic model to a model with conventional CHD risk factors as predictors, a similar approach was employed to build the model on the training data and subsequently test it on the test dataset. The filtering and modeling steps are summarized in Fig 1. Also, net reclassification improvement analysis[27] was performed to compare the improvement in classification using the integrated model.

Additionally, to compare our ensemble approach to a single RF model, an alternative RF model was developed in R using the randomForest package. The *“strata”* and *“sampsize”* arguments were used to perform stratified sampling of the minority class. This is a simpler implementation of the undersampling ensemble approach described above. The number of trees (ntree) parameter of this alternative RF classifier was tuned. The same n = 1,545 training set and n = 142 testing set were used to train and test this classifier.

### Statin use analysis

To identify statin use associated genome-wide DNA methylation changes, linear regression analyses were conducted using data from the 1,545 individuals in the training set in R V3.1.2 as delineated in Eq (2):

$${Meth}_{i}\;\sim \;Age+Gender+Batch+CHD+Statin\;Use$$ (2)

The association between DNA methylation and statin use was determined while controlling for age, gender, DNA methylation laboratory batch effects and CHD status. Bonferroni correction for multiple comparisons at a genome-wide α = 0.05 was performed for every regression analysis.[21] A total of 472,822 independent tests were conducted and therefore, only those with a nominal p-value <1e-07 (0.05/472822) were considered to be significantly associated at the genome-wide level.

## Results

The clinical characteristics of the 1,545 and 142 subjects used for training and testing, respectively, in this study are given in Table 1. There were more females (n = 851/~55%) than males (n = 694/~45%) in the training set, but more males (n = 88/~62%) than females (n = 54/~38%) in the test set. They were all of Northern European ancestry. A total of 115 males (~17%) and 58 females (~7%) in the training set, and 22 females (~41%) and 49 males (~56%) in the test set were diagnosed with symptomatic CHD. Those with symptomatic CHD on average tended to be older than those without symptomatic CHD. Also, on average, males in the training set were older than those in the testing set, while average age of females were more comparable between both sets.

**Table 1:** Summary of variables. The demographics and CHD risk factors of the 1,545 and 142 individuals included in the training and testing sets, respectively.

	Training		Testing	
	CHD	No CHD	CHD	No CHD
**Gender (count)**				
Male	115	579	49	39
Female	58	793	22	32
**Age (years)**				
Male	71.1±7.4	66.4±8.5	67.5±8.4	59.6±9.2
Female	73.0±8.7	66.4±8.6	72.5±9.0	64.6±10.8
**Total Cholesterol (mg/dL)**				
Male	154±33	176±33	141±25	191±32
Female	172±35	199±36	180±41	187±35
**HDL Cholesterol (mg/dL)**				
Male	45±12	50±14	46±11	51±15
Female	59±17	65±19	62±18	61±18
**HbA1c (%)**				
Male	6.0±0.9	5.7±0.8	5.9±0.9	5.9±1.4
Female	6.0±0.9	5.7±0.5	6.3±1.0	6.0±1.0
**SBP (mmHg)**				
Male	128±19	130±17	124±19	127±17
Female	135±18	129±18	136±17	129±15
**Use Statin (count)**				
Male	94	228	1	3
Female	44	254	0	0
**cg05575921 (z-score)**				
Male	-0.15±1.19	-0.07±1.05	-0.46±1.43	-0.26±1.12
Female	-0.12±1.11	0.08±0.92	0.10±1.12	-0.13±0.93
**Smoker (count)**				
Male	12	39	6	7
Female	2	64	1	4

The mean and standard deviations of other conventional risk factors are also summarized in Table 1. While HDL cholesterol, SBP and HbA1c were comparable between individuals in the training and testing sets, total cholesterol was not. This could be attributed to the use of statins as outlined in Table 1. Also, a well-known risk factor for CHD is smoking. As shown in Table 1, based on self-reported current smoking status, more individuals without CHD reported smoking than those with CHD. However, since self-reported smoking status is known to be less reliable, including in cardiac patients[28, 29], we also include the epigenetic smoking biomarker, cg05575921[30], in Table 1.

Regression of CHD to genome-wide DNA methylation using data from the 1,545 individuals in the training set resulted in 11,497 significant methylation sites (2.4%) after Bonferroni correction for multiple comparisons. The top 30 sites are shown in Table 2, while all sites are shown in S1 File. All annotations (Illumina) are based on genome build 37 and in S1 File, additional annotation (uncorrected p-values and mapinfo) are provided for all 11,497 sites.

**Table 2:** DNA methylation and CHD status. Top 30 significant methylation sites associated with symptomatic CHD after Bonferroni correction for multiple comparisons using training set data.

CpG	Beta	Gene	Chr	Position	Island Status	Corrected p-value
cg26910465	6.48E-01	ADAL	15	TSS200	Island	8.01E-18
cg13567813	6.60E-01	NR1H2	19	TSS200	Island	2.05E-17
cg09238957	5.98E-01	ORC6L	16	TSS200	Island	7.97E-17
cg04099813	6.12E-01	TSSC4	11	TSS1500	S_Shore	1.45E-16
cg07546106	6.29E-01	TAP2	6	5’UTR	N_Shore	2.40E-16
cg20808462	6.01E-01	HAUS3	4	5’UTR	Island	5.42E-16
cg16968115	5.92E-01	WDTC1	1	TSS200	Island	1.25E-15
cg24475210	5.84E-01	MRFAP1	4	TSS200	Island	1.26E-15
cg03031660	5.84E-01	MRPS7	17	1stExon	Island	1.45E-15
cg22605179	5.97E-01	EWSR1	22	5’UTR	Island	3.81E-15
cg02357877	5.71E-01	GBAS	7	TSS1500	Island	4.04E-15
cg22111723	5.65E-01		13		Island	4.57E-15
cg06117184	5.67E-01	CKAP2L	2	1stExon	Island	4.87E-15
cg07478100	5.85E-01	MIS12	17	TSS1500	Island	5.36E-15
cg15318396	5.83E-01		21		Island	5.52E-15
cg00544901	5.76E-01	RPS11	19	TSS1500	Island	5.62E-15
cg24478630	5.88E-01	MOGS	2	TSS200	S_Shore	5.65E-15
cg04022019	5.90E-01	DCAF13	8	1stExon	Island	5.86E-15
cg12124516	5.81E-01	MCM6	2	TSS200	Island	6.41E-15
cg20935862	5.96E-01	C9orf41	9	TSS1500	Island	6.62E-15
cg07377675	6.00E-01	USP1	1	TSS200	Island	7.79E-15
cg07734253	5.83E-01	CORO1A	16	TSS1500	N_Shore	8.16E-15
cg03699307	5.94E-01	GABARAPL2	16	TSS1500	Island	8.44E-15
cg17360140	5.79E-01	C4orf29	4	TSS1500	Island	9.10E-15
cg25632648	6.06E-01	KCTD21	11	TSS200	Island	9.83E-15
cg06339248	5.83E-01	ZDHHC5	11	5’UTR	Island	1.10E-14
cg24275354	6.24E-01	NDUFA10	2	Body	N_Shore	1.52E-14
cg25261764	5.93E-01	NARS	18	1stExon	Island	1.69E-14
cg14172283	5.83E-01	TOMM5	9	1stExon	Island	2.00E-14
cg01089095	5.69E-01	CHCHD1	10	TSS200	Island	2.02E-14

### Integrated genetic-epigenetic Random Forest analyses

**Fig 2:**
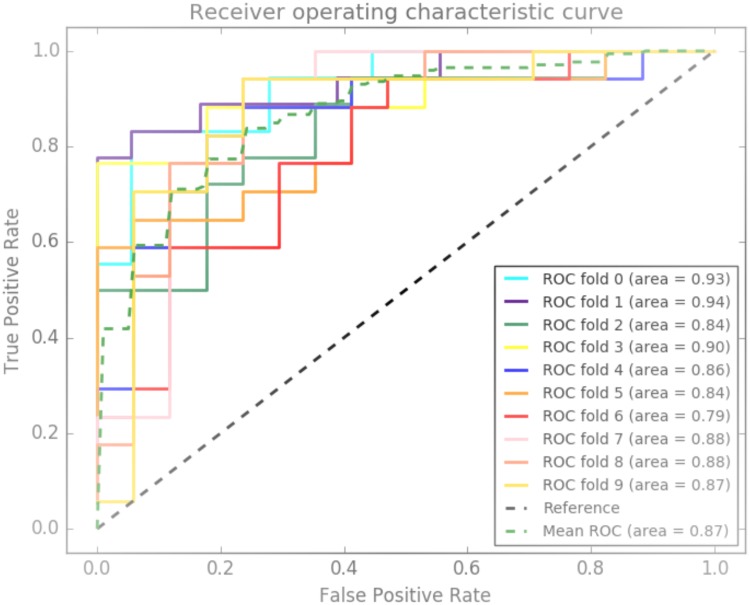
Integrated genetic-epigenetic model ROC curve. The Receiver Operating Characteristic curves of the integrated genetic-epigenetic model with the largest average 10-fold cross-validation area under the curve value.

Eight RF models were built on the eight sub-datasets consisting of genetic, epigenetic, age and gender data from the 1,545 subjects in the training dataset. Standard scikit-learn RF parameters were used to determine the important SNPs and DNA methylation loci. Based on the average accuracy and AUC of the eight classifiers and the information gain criteria of each variable, four CpG sites (cg26910465, cg11355601, cg16410464 and cg12091641), two SNPs (rs6418712 and rs10275666), age and gender were retained for prediction. Using the tuned parameters, all eight models were re-fitted to the training dataset. The performance metrics (mean and standard deviations) of these stratified 10-fold cross-validated models are shown in Table 3. As depicted in this table, the accuracy ranges from 70–80% between these eight models, which is between a 20–30% increase from the 50% accuracy baseline. More importantly, the sensitivity of the model ranged from 70–82%, while the specificity ranged from 70–79%. The ROC AUC of the eight models ranged from 0.77–0.87. The 10-fold ROC AUC of the best performing model (model 7) is shown in Fig 2. All eight models were saved for testing on the test dataset (n = 142).

**Table 3:** Integrated genetic-epigenetic training metrics. The 10-fold cross-validation performance metrics of the eight integrated genetic-epigenetic models within the ensemble on the training set.

Model	Accuracy	AUC	Sensitivity	Specificity
1	0.78±0.09	0.82±0.09	0.79±0.12	0.77±0.08
2	0.75±0.05	0.83±0.06	0.78±0.10	0.72±0.08
3	0.79±0.05	0.85±0.07	0.83±0.07	0.76±0.08
4	0.78±0.07	0.84±0.07	0.79±0.12	0.76±0.07
5	0.75±0.06	0.78±0.06	0.70±0.09	0.79±0.09
6	0.70±0.05	0.77±0.05	0.70±0.12	0.70±0.10
7	0.80±0.06	0.87±0.04	0.82±0.08	0.77±0.07
8	0.78±0.06	0.85±0.05	0.82±0.07	0.74±0.08

An ensemble of the eight models was used to perform CHD classification in the test dataset. An individual was classified as having CHD if at least four of the eight models voted in favor of CHD. Of the 142 individuals (71 with and 71 without symptomatic CHD) in the test dataset, the CHD status of 110 individuals was predicted correctly, resulting in a test accuracy of 77.5%. The confusion matrix of the prediction is shown in Table 4. The test set sensitivity and specificity of the ensemble was 0.75 and 0.80, respectively. When the ensemble was tested on the full test set of n = 669, the sensitivity held and the specificity increased to 0.82.

**Table 4:** Testing of integrated genetic-epigenetic ensemble. The confusion matrix of the integrated genetic-epigenetic ensemble of eight models on the test dataset consisting of 142 individuals.

	Predicted	
**TRUE**	**CHD absent**	**CHD present**
**CHD absent**	57	14
**CHD present**	18	53

To understand the variance of each of the four CpG sites captured by the conventional risk factors, using training set data, regression analysis was performed and the results are summarized in S2 File. Briefly, as shown in this supporting file, these four CpG sites are significantly associated with age, gender, total cholesterol, cg05575921 smoking or HbA1c. This suggests that each CpG site can be linked to risk factors that commonly assessed. Furthermore, to determine if relatedness may have contributed to the observed performance of the models, additional analysis and results are described in S3 File. Results provided in the form of visualization and logistic regression in this supporting file suggest that relatedness did not influence the prediction accuracy of the model in the test set.

Additionally, the list of genome-wide DNA methylation signatures associated with statin use, controlling for age, gender, batch and CHD status, which was obtained using training set data was compared to the list of the four CpG sites. As shown in Table 5, after correction for multiple comparisons, statin use was significantly associated with seven CpG sites. However, this list did not include any of the four CpGs contained within the integrated genetic-epigenetic model. The nominal p-values prior to correction for multiple comparisons of the four CpG sites were 0.002, 0.908, 0.109 and 0.015 for cg26910465, cg11355601, cg16410464 and cg12091641, respectively.

**Table 5:** DNA methylation and statin use. All significant methylation sites associated with statin use after Bonferroni correction for multiple comparisons using training set data.

CpG	Beta	Gene	Chr	Position	Island Status	Corrected p-value
cg17901584	-4.59E-01	DHCR24	1	TSS1500	S_Shore	3.53E-12
cg06500161	4.43E-01	ABCG1	21	Body	S_Shore	9.30E-11
cg05119988	-3.30E-01	SC4MOL	4	5’UTR	S_Shelf	5.22E-04
cg19751789	-3.23E-01	LDLR	19	TSS200	N_Shore	1.54E-03
cg27243685	3.01E-01	ABCG1	21	Body	S_Shelf	1.87E-02
cg01185530	-2.58E-01	DNAJC3	13	5’UTR	Island	2.81E-02
cg11072882	-2.74E-01	FAM47E	4	Body	Island	4.97E-02

### Conventional CHD risk factor model

**Fig 3:**
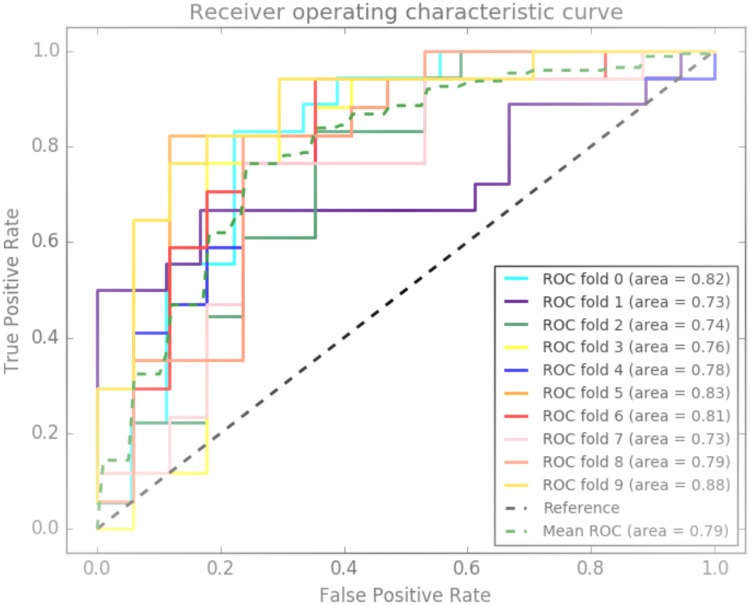
Conventional risk factors model ROC curve. The Receiver Operating Characteristic curves of the conventional risk factors model with the largest average 10-fold cross-validation area under the curve value.

To compare the performance of our integrated genetic-epigenetic model to the performance of conventional CHD risk factors in predicting CHD status, another eight RF models were built using age, gender, SBP, HbA1c, total cholesterol, self-reported smoking and HDL cholesterol as predictors. Again, using tuned parameters, the eight RF models were built on the training dataset and tested on the test dataset. The performance metrics (mean and standard deviations) of the eight models are summarized in Table 6. Accuracies of these models on their respective training datasets ranged from 70–76%, while the sensitivity and specificity ranges were 67–74% and 72–79%, respectively. The range of the ROC AUC was 0.72–0.79. While the accuracy and specificity is quite comparable with the integrated genetic-epigenetic models, the conventional risk factors models underperformed with respect to sensitivity and ROC AUC. The 10-fold ROC AUC of the best performing model (model 7) among the eight models is shown in Fig 3.

**Table 6:** Conventional risk factor training metrics. The 10-fold cross-validation performance metrics of the eight conventional risk factor models within the ensemble on the training set.

Model	Accuracy	AUC	Sensitivity	Specificity
1	0.73±0.03	0.77±0.05	0.71±0.07	0.75±0.10
2	0.73±0.07	0.75±0.08	0.74±0.08	0.72±0.09
3	0.75±0.07	0.79±0.06	0.73±0.12	0.77±0.10
4	0.70±0.06	0.75±0.08	0.68±0.10	0.72±0.07
5	0.70±0.06	0.72±0.08	0.67±0.09	0.73±0.10
6	0.71±0.10	0.75±0.10	0.68±0.14	0.75±0.10
7	0.76±0.04	0.79±0.05	0.73±0.11	0.79±0.09
8	0.71±0.10	0.76±0.12	0.68±0.15	0.75±0.11

When the ensemble of the eight models was tested on the test dataset, the test accuracy was 65.4%, which is approximately 12% less than that of our integrated genetic-epigenetic ensemble. The confusion matrix is shown in Table 7. However, the more important metric is the sensitivity since it shows the degree to which a person with CHD is classified correctly. The sensitivity on the test dataset was only 42%, which is 33% less than that of our integrated genetic-epigenetic ensemble. However, the specificity of the conventional risk factor ensemble was 0.89. When the ensemble was tested on the full test set of n = 669, the sensitivity held and the specificity increased to 0.92. The net reclassification improvement using the integrated genetic-epigenetic model instead of this conventional risk factors model was 0.25.

**Table 7:** Testing of conventional risk factors ensemble. The confusion matrix of the conventional risk factors ensemble of eight models on the test dataset consisting of 142 individuals.

	Predicted	
TRUE	CHD absent	CHD present
**CHD absent**	63	8
**CHD present**	41	30

### Alternative Random Forest model

To determine if our ensemble approach consisting of eight models performs better than a single RF model, as described in the methods, one RF model that includes stratified sampling based on the minority class was built in R. The results are discussed in S3 File. Briefly, these results suggest that the ensemble approach results in higher prediction sensitivity than the single stratified modeling approach.

## Discussion

A better understanding of the relationship of epigenetic changes to the pathogenesis of cardiovascular diseases is essential for the development of improved diagnostics and therapeutics. In our analysis to uncover epigenetic signatures associated with CHD, cg26910465 from the *ADAL* gene was the most significantly differentially methylated with respect to CHD status. This gene is part of the adenosine deaminase family.[31] Previous studies have examined the relationship between genes in the adenosine deaminase gene family and CHD [32–34], given the important cardioprotective role of adenosine.[35] The inclusion of epigenetic signatures is particularly attractive given the challenges and limitations in using multivariate risk models consisting of conventional risk factors to predict the risk for CHD. For instance, the Framingham Risk Score for CHD [36] which incorporates conventional risk factors and was developed using the FHS cohort consisting of individuals of European ancestry, performed well for white men and women, but hardly generalized to all other ethnic groups. Specifically, in a study that validated this algorithm in an ethnically diverse cohort, the prediction model held for black men and women, but overestimated risk of Japanese Americans, Hispanic men and Native American women.[37] Hence, there is a need for models that can be used for all members of our society.

One plausible reason for the lack in generalizability is the possible confounding effects of genetic variation. The concept of the potential for genetic confounding of epigenetic signal is widely accepted.[38] Therefore, the goal of our study was to integrate genetic and epigenetic data to develop a classifier to predict symptomatic CHD as a step towards demonstrating the ability to use this integrated approach for future risk models as an alternative to existing algorithms. This approach that mines predictive signal from large and complex genetic and epigenetic datasets is made possible by the advancements in high performance computing systems. Computational techniques such as machine learning have been successfully employed in the fields of genomics and epigenomics.[39, 40] While logistic regression is the commonly used method for developing binary classification models in medical applications and have been used to analyze microarray data [41], it lacks the ability to capture implicit complex nonlinear relationships. Hence, algorithms capable of detecting complex relationships such as interactions between genetic variation and DNA methylation have an added advantage. In our study, the use of a Random Forest ensemble allowed for a highly accurate, sensitive and specific classification of individuals with CHD. However, since some genetic risk variants co-sort with ethnic background and may not map to pathways associated with conventional risk factors, it will be necessary to build, test and extend these Random Forest approaches using subjects from all ethnic groups to develop more generalizable prediction tools.[42]

While a similar integrated genetic and epigenetic study is not available for comparison, our integrated model clearly outperforms the classifier that uses the conventional risk factors. The conventional risk factors model demonstrates the limited predictive value of these risk factors as indicated by a number of studies.[43–45] In a study consisting of over 2000 older black and white adults, the Framingham risk score, which uses conventional risk factors, was only capable of distinguishing those who experienced a CHD event versus those who did not after an eight year follow-up at a C-index of 0.577 and 0.583 in women and men, respectively.[46] The conventional risk factors may not perform as well due to hourly variations of factors such as serum cholesterol level and the use of a single blood pressure measurement instead of an average recorded throughout the day.[47, 48]

As demonstrated in this manuscript, there are several approaches to building classifiers. In comparing the two methods delineated in this manuscript, the ensemble model performed better than the single RF model (S4 File) with respect to sensitivity and vice versa for specificity. Our reason to favor a model with higher sensitivity is simple. For the classification of diseases such as CHD, a false positive would require further testing but a false negative result could be detrimental to the patient. However, a test with high sensitivity and specificity is ideal. To achieve that, a larger sample consisting of diverse ethnic groups encompassing both genders is required. Also, while we used the RF algorithm, there are many other algorithms such as Support Vector Machines that can be used as the algorithm underlying a classifier. Nevertheless, our RF model clearly shows non-linearity between methylation sites and SNPs as depicted in the partial dependence plots.

It is important to realize that the combination of methylation sites and SNPs in our ensemble (cg26910465, cg11355601, cg16410464, cg12091641, rs6418712 and rs10275666) is only one of large number possible combinations that could be highly predictive. The first three CpG sites, cg26910465, cg11355601, and cg16410464, map to genes *ADAL*, *JOSD1*, and *CCT6P1*, respectively, while cg12091641 does not map to a specific gene. *ADAL* has been described above. The gene, *JOSD1*, has been reported to be upregulated in peripheral blood mononuclear cells in those with asymptomatic myocardial dysfunction.[49] *CCT6P1* is the pseudogene of the Chaperonin Containing TCP1 (CCT) Subunit 6 gene. The protein of this gene is one of the subunits of the CCT complex. While *CCT6P1* has not been directly implicated in CHD, the CCT complex has been shown to interact with LOX-1, a receptor involved in atherogenesis.[49] The first tag SNP, rs6418712, maps to an X chromosome locus (giving us absolute gender), containing a poorly characterized transcript whose highest expression is in blood; NHS, which is an actin remodeling regulator, and several microRNAs. The second, rs10275666, is in very tight linkage disequilibrium (LD) with *HUS1*, a gene involved in CVD related oxidative DNA repair [50–53] and in strong LD with MIR590, a putative microRNA biomarker for CVD and established regulator of cardiac development/regeneration.[54–56]

Based on the permutation results (Fig 2 in S4 File), we demonstrate that the variable reduction step undertaken to enrich for highly predictive methylation and SNP probes provide an edge with respect to sensitivity. However, the current ensemble was built using data only from individuals of Northern European extraction. Because the current set of markers used in our ensemble may not be as highly predictive in subjects of other ethnicities or cultures, the potential combinations will have to be parsed in order to provide a highly predictive yet generalizable classifier for all members of our society. Nevertheless, as shown in S2 File, the methylation sites included in our model can be linked to conventional risk factors, suggesting that a portion of the variance of these sites can be explained by a combination of risk factors.

Our analyses did not take into account the possible effects of medications. This is notable because the current armamentarium of cholesterol lowering agents can have a dramatic effect on the levels of certain risk factors, such as serum cholesterol, that are associated with risk for CHD. Indeed, the presence of these medications may be the reason why the serum cholesterol level is actually lower in those in the training set who have CHD than in those in the training set without CHD. Unfortunately, it is very difficult to incorporate these types of data into the current analytical approach for several reasons. Even if the subjects self-report of prescriptions were accurate, critical information needed to account for their effects, such as medication compliance and the treatment history length are not available. In the future, having data such as “pill count” and serum drug level information will be critical if we are to fully understand the effect of medical interventions on epigenetic signatures used for disease prediction.

Although it is possible that a portion of model variance is secondary to the effects of medications, we will note that to date, an epigenetic signature of statins on peripheral DNA methylation has not been identified. However, our statin use analysis show that none of the four methylation probes in our integrated genetic-epigenetic model are significantly associated with statin use. This may be due to 35% of the non-CHD subjects also taking statins. Nevertheless, it is without question that future studies must be wary of the potential for medication effects to alter predictive capacity.

There are several other limitations in our study. First, our study includes only individuals of European ancestry. However, the incorporation of genetic variation in our model allows for the generalizability between ethnic groups. Nevertheless, additional studies are required to demonstrate this. Second, testing of the ensemble models were not performed on an independent cohort. Third, while our approach predicts symptomatic CHD, the goal is to use this study as a proof-of-concept towards building a multivariate model capable of forecasting risk for a future CHD event. Extensive exploration in prospectively biosampled cohorts will be necessary to achieve that goal. Yet, it is important to note that this integrated genetic-epigenetic approach has its advantages. The use of conventional risk factors in calculating risk requires cumbersome testing procedures, the collection of considerable amounts of blood and multiple lab tests. Conceivably, the need for these often cumbersome tests and procedures will be greatly reduced by using a single genetic-epigenetic assay procedure that uses a microgram or less of DNA. More importantly, the pathways associated with specific epigenetic loci with high predictive value could be very useful in guiding therapeutic interventions, management of risk factors and monitoring efficacy of treatments and lifestyle modifications.

## Supporting information

S1 File All CHD associated significant CpG sites after correction for multiple comparisons. (TXT)

S2 File Variance of each CpG site captured by conventional risk factors. (PDF)

S3 File Contribution of relatedness to performance of models. (PDF)

S4 File Performance of Random Forest model with stratified sampling based on the minority class. (PDF)
