# Supplementary material for: Integrated genetic and epigenetic prediction of coronary heart disease in the Framingham Heart Study
Source: PLoS One. 2018 Jan 2;13(1):e0190549. doi: 10.1371/journal.pone.0190549 (PMC5749823; doi:10.1371/journal.pone.0190549)
Supplement: S2 File — (PDF) [file pone.0190549.s002.pdf]

To determine the association between the four CpG sites included in the prediction model and conventional risk factors, the following regression was performed for each of the CpG site:

$$CpG \sim Age + Gender + cg05575921 + SBP + HDL\ cholesterol + total\ cholesterol + HbA1c$$

The regression results for cg26910465, cg11355601, cg16410464 and cg12091641 are shown in Tables 1 through 4, respectively. The R-squared values for these sites were 13.52%, 11.72%, 11.21% and 13.04%, respectively.

Table 1. Regression parameters for cg26910465

**Variable**	**Beta**	**Standard Error**	**t-value**	**p-value**
**Age**	2.41e-02	2.95e-03	8.15	7.57e-16
**Gender**	-4.30e-01	5.38e-02	-7.99	2.58e-15
**cg05575921**	3.31e-02	2.40e-02	1.38	0.17
**SBP**	-2.09e-03	1.43e-03	-1.46	0.14
**HDL cholesterol**	-7.23e-04	1.54e-03	-0.47	0.64
**Total cholesterol**	-2.99e-03	7.27e-04	-4.11	4.20e-05
**HbA1c**	7.90e-02	3.62e-02	2.18	0.03

Table 2. Regression parameters for cg11355601

**Variable**	**Beta**	**Standard Error**	**t-value**	**p-value**
**Age**	2.08e-02	2.98e-03	6.98	4.37e-12
**Gender**	-4.13e-01	5.44e-02	-7.59	5.43e-14
**cg05575921**	6.37e-02	2.42e-02	2.63	8.71e-03

**SBP**	-1.17e-03	1.44e-03	-0.81	0.42
**HDL cholesterol**	-7.46e-04	1.55e-03	-0.48	0.63
**Total cholesterol**	-2.72e-03	7.34e-04	-3.71	2.16e-04
**HbA1c**	7.03e-02	3.66e-02	1.92	5.51e-02

Table 3. Regression parameters for cg16410464

**Variable**	**Beta**	**Standard Error**	**t-value**	**p-value**
**Age**	2.17e-02	2.99e-03	7.24	7.15e-13
**Gender**	-3.45e-01	5.45e-02	-6.32	3.35e-10
**cg05575921**	9.12e-02	2.43e-02	3.75	1.82e-04
**SBP**	-7.71e-04	1.45e-03	-0.53	0.59
**HDL cholesterol**	-9.66e-04	1.56e-03	-0.62	0.53
**Total cholesterol**	-2.62e-03	7.36e-04	-3.56	3.86e-04
**HbA1c**	9.68e-02	3.67e-02	2.64	8.50e-03

Table 4. Regression parameters for cg12091641

**Variable**	**Beta**	**Standard Error**	**t-value**	**p-value**
**Age**	2.14e-02	2.96e-03	7.24	7.21e-13
**Gender**	-3.61e-01	5.39e-02	-6.69	3.04e-11
**cg05575921**	1.11e-01	2.40e-02	4.62	4.17e-06
**SBP**	-1.41e-03	1.43e-03	-0.98	0.33
**HDL cholesterol**	-1.25e-03	1.54e-03	-0.81	0.42

**Total cholesterol**	-3.18e-03	7.28e-04	-4.37	1.30e-05
**HbA1c**	1.10e-01	3.63e-02	3.04	2.41e-03
