# Supplementary material for: Integrated genetic and epigenetic prediction of coronary heart disease in the Framingham Heart Study
Source: PLoS One. 2018 Jan 2;13(1):e0190549. doi: 10.1371/journal.pone.0190549 (PMC5749823; doi:10.1371/journal.pone.0190549)
Supplement: S3 File — (PDF) [file pone.0190549.s003.pdf]

To examine if the prediction accuracy of those in the n=142 test set was a function of their relatedness (i.e. IBD), the maximum IBD of individuals and their respective prediction accuracy were visualized. Among the 142 individuals in the test set, based on their IBD values shown in Fig 1, 130 and 12 individuals are first-degree and second-degree relatives, respectively, to those in the training set. As seen in Fig 1, there appears to be no obvious relationship between IBD and whether an individual's symptomatic CHD status was predicted correctly. In other words, those who were predicted correctly and incorrectly did not cluster into two distinct IBD groups. To further demonstrate this, logistic regression was performed to determine if a significant relationship was present between IBD (continuous) and prediction accuracy outcome (categorical). A p-value of 0.95 suggested that relatedness did not influence the obtained prediction accuracy.

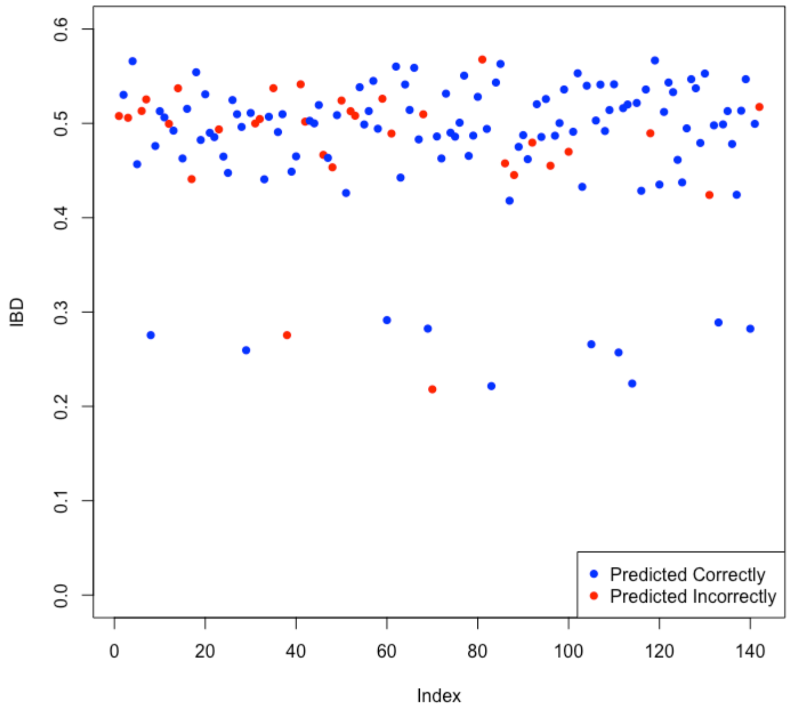

Fig 1. The prediction accuracy and IBD of the 142 individuals in the test set
