# Supplementary material for: Integrated genetic and epigenetic prediction of coronary heart disease in the Framingham Heart Study
Source: PLoS One. 2018 Jan 2;13(1):e0190549. doi: 10.1371/journal.pone.0190549 (PMC5749823; doi:10.1371/journal.pone.0190549)
Supplement: S4 File — (PDF) [file pone.0190549.s004.pdf]

The single stratified Random Forest (RF) model included the same four CpGs, two SNPs, age and gender. The classifier was tuned and the classifier with the largest sensitivity was chosen (ntree=500). The training accuracy, AUC, sensitivity and specificity of this model were 82%, 0.83, 0.68 and 0.83, respectively. While the accuracy, AUC and specificity of this model is comparable to our ensemble model, clearly, the ensemble model provides better sensitivity. When tested on the test set, the single RF model performed with an accuracy, sensitivity and specificity of 76%, 0.66 and 0.86, respectively, demonstrating the increased sensitivity but not specificity provided by the ensemble approach. The comparison between this alternative approach and the ensemble approach is being done on the basis of sensitivity rather than specificity is because, given the application of the classifier, predicting CHD, it is rather important to maximize true positives than true negatives. In other words, the impact of having a false negative is much higher than a false positive.

While age and gender were included because they are the two non-modifiable risk factors of CHD, we re-fitted the single RF model without age and gender to demonstrate that the performance is not driven solely by these two factors. Without age and gender in the model, the training accuracy, AUC, sensitivity and specificity were 81%, 0.80, 0.65 and 0.83, respectively. On the test dataset, this model performed with an accuracy, sensitivity and specificity of 78%, 0.68 and 0.89, respectively. Therefore, age and gender are not single handedly responsible for the performance of the integrated genetic-epigenetic model. Using conventional risk factors from the training dataset, this alternative RF model performed with an accuracy, AUC, sensitivity and specificity of 77%, 0.77, 0.60 and 0.79, respectively. On the test dataset, it performed with an accuracy, sensitivity and specificity of 69%, 0.61 and 0.77, respectively.

This genetic-epigenetic model was also used to show that the use of a RF model provides an added advantage in capturing possible GxM and MxM interactions, as depicted by the partial dependence plots in Fig 1. Finally, permutation of DNA methylation sites and genotypes was performed to compare the performance of a model consisting of four randomly chosen CpG sites and two randomly chosen SNPs using the training dataset to our integrated model and the conventional risk factor model. A two-dimensional histogram of sensitivities and specificities of 10,000 permutations are shown in Fig 2. The largest sensitivity and specificity among these permutations were 0.62 and 0.87, respectively. As shown in this figure, the training sensitivity and specificity of the single conventional risk factors model (CRF Model) of 0.60 and 0.79, respectively, falls well within the sensitivity and specificity of the permutations. The training sensitivity and specificity of the single integrated genetic-epigenetic model with age and gender (GE Model) of 0.68 and 0.83, respectively, and without age and gender (GE Model 2) of 0.65 and 0.83, respectively, suggest that sensitivity but not the specificity falls outside the permuted values.

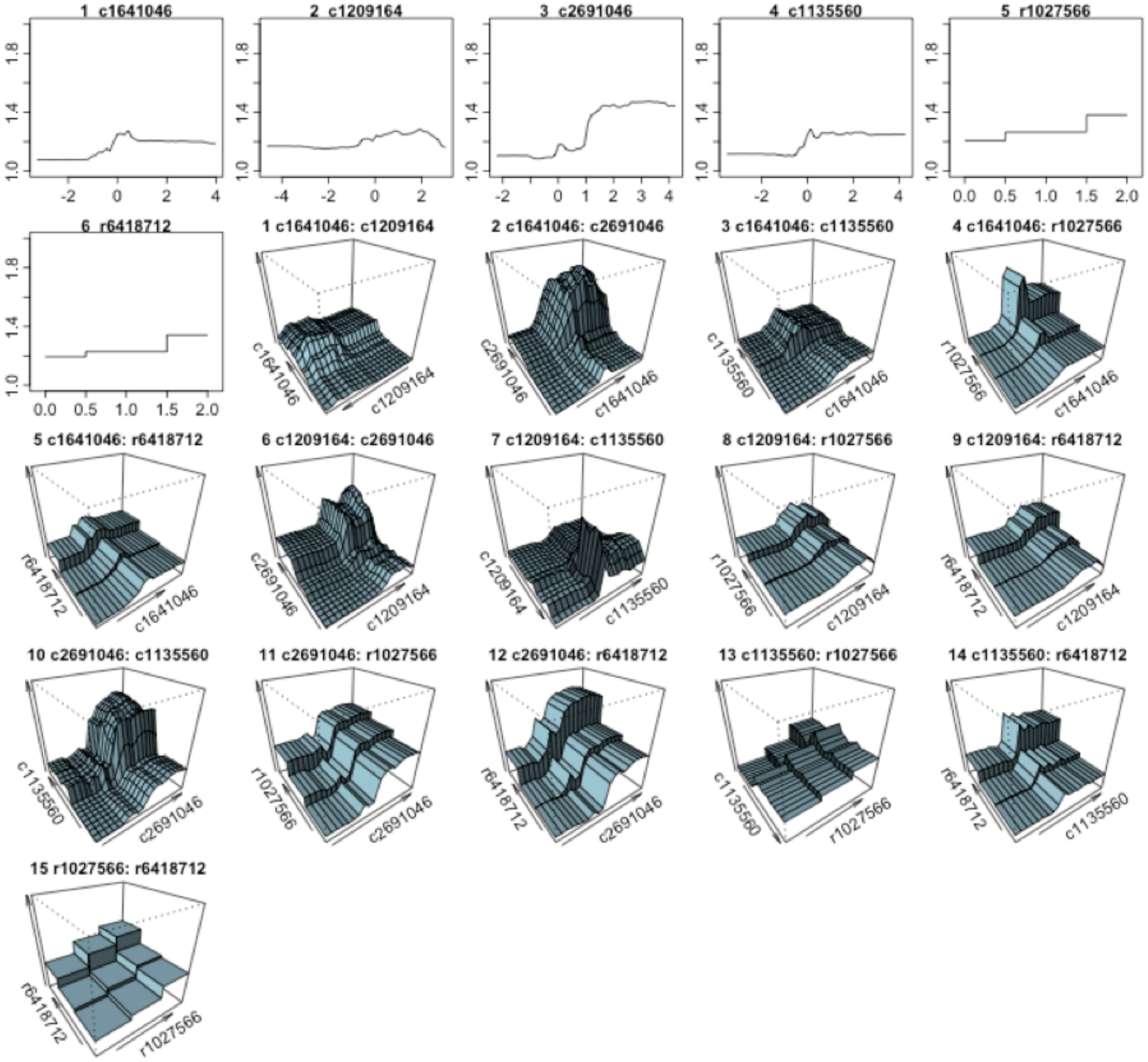

Fig 1. Partial dependence plots. The partial dependence plots between each DNA methylation site and each SNP included in the integrated genetic-epigenetic model.

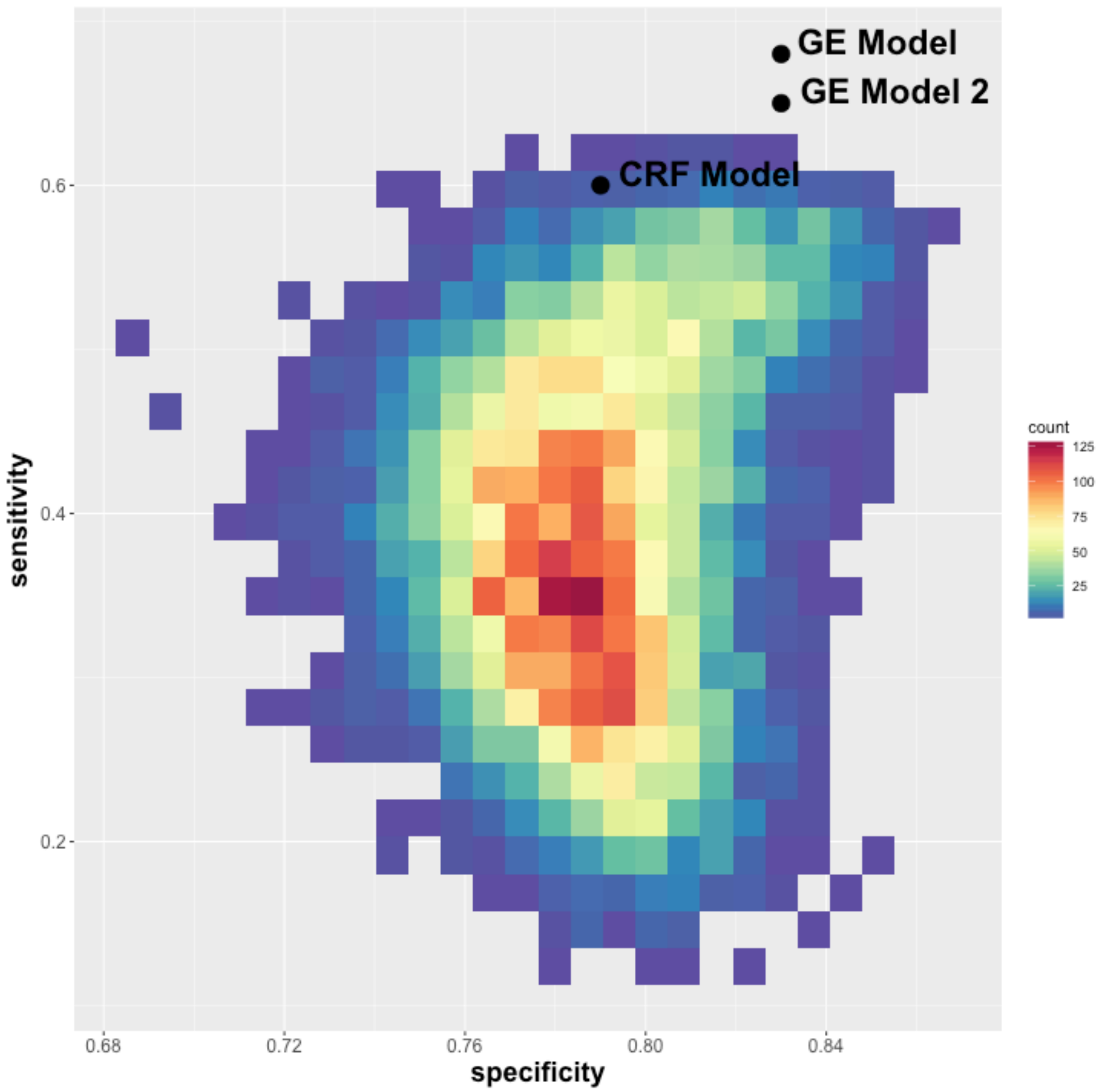

Fig 2. Two-dimensional histogram of sensitivity and specificity of 10,000 permutations of DNA methylation sites and SNPs.
